# Evaluation of the bioequivalence and food effect on the bioavailability of CC-486 (oral azacitidine) tablets in adult patients with cancer

**DOI:** 10.1007/s00280-020-04037-9

**Published:** 2020-02-08

**Authors:** Hani M. Babiker, Mohammed Milhem, Joseph Aisner, William Edenfield, Dale Shepard, Michael Savona, Swaminathan Iyer, Maen Abdelrahim, C. L. Beach, Barry Skikne, Eric Laille, Kao-Tai Tsai, Thai Ho

**Affiliations:** 1 University of Arizona Comprehensive Cancer Center, Tucson, AZ USA; 2 University of Iowa Hospital, Iowa City, IA USA; 3 Rutgers Cancer Institute of New Jersey, New Brunswick, NJ USA; 4 Greenville Health System, Greenville, SC USA; 5 Cleveland Clinic, Cleveland, OH USA; 6 Vanderbilt University Medical Center, Nashville, TN USA; 7 MD Anderson Cancer Center, Houston, TX USA; 8 Houston Methodist Hospital, Houston, TX USA; 9 Celgene Corporation, Summit, NJ USA; 10 University of Kansas Medical Center, Kansas City, KS USA; 11 Mayo Clinic, Phoenix, AZ USA

**Keywords:** Oral azacitidine, CC-486, Pharmacokinetics, Bioequivalence

## Abstract

**Purpose:**

CC-486 is an oral formulation of azacitidine that allows for extended dosing schedules to prolong azacitidine exposure to malignant cells and maximize clinical activity. CC-486 300 mg daily, administered for 14 or 21 days of 28-day treatment cycles, is currently under investigation in two ongoing phase III trials. The 300-mg daily dose in these studies is administered as two 150-mg tablets (Formulation A).

**Methods:**

We evaluated the bioequivalence of one 300-mg CC-486 tablet (Formulation B) with Formulation A and food effect on Formulation B, in adult patients with cancer in a 2-stage crossover design study.

**Results:**

The ratios of the geometric means of the maximum azacitidine plasma concentration (*C*_max_) and of the area under the plasma concentration–time curve from time 0 extrapolated to infinity (AUC_*∞*_) were 101.5% and 105.7%, demonstrating the bioequivalence of Formulations A and B. Formulation B was rapidly absorbed under fasted and fed conditions. The geometric mean of *C*_max_ was significantly decreased by ~ 21% in the fed state. Median *T*_max_ was reached at 2 h and 1 h post-dose in fed and fasted states, respectively (*P* < 0.001). Nevertheless, systemic drug exposure (AUC) in fed and fasted states was within the 80–125% boundaries of bioequivalence and differences in *C*_max_ and *T*_max_ are not expected to have a clinical impact.

**Conclusion:**

The single 300-mg CC-486 tablet was bioequivalent to two 150-mg tablets, which have shown to be efficacious and generally well-tolerated in clinical trials, and can be taken with or without food.

**Electronic supplementary material:**

The online version of this article (10.1007/s00280-020-04037-9) contains supplementary material, which is available to authorized users.

## Introduction

Azacitidine is a cytidine nucleoside analog and DNA methyltransferase inhibitor (DNMTi) used in the treatment of patients with various malignancies. Injectable azacitidine 75 mg/m^2^/day, administered intravenously (IV) or subcutaneously (SC) for 7 days per 28-day treatment cycle, is approved in several countries for treatment of myelodysplastic syndromes (MDS) and acute myeloid leukemia (AML) [1, 2]. Azacitidine has also been investigated in a variety of other hematologic malignancies and solid tumors [3–6]. In addition to direct cytotoxicity of proliferating malignant cells, azacitidine is incorporated into both RNA and DNA (in human AML KG1a cells, the RNA:DNA incorporation ratio was 65:35 [7]) and reduces hypermethylation in promoter regions of DNA, leading to re‐expression of tumor suppressor genes and promoting differentiation of hematopoietic progenitor cells [7–9]. Azacitidine has a short plasma half-life, and DNA incorporation of azacitidine is S-phase restricted [10]. DNA hypomethylation is highest during the first half of each injectable azacitidine treatment cycle, but returns to baseline levels by the end of the cycle [11].

CC-486 is an oral formulation of azacitidine that allows for extended dosing schedules to prolong azacitidine exposure and thus maximize hypomethylating effects [12, 13]. Early clinical studies showed CC-486 to be bioavailable, well-tolerated, and clinically active in patients with MDS, AML, or chronic myelomonocytic leukemia (CMML) [11, 12, 14]. When administered for 7 days per treatment cycle, global DNA hypomethylation with CC-486 was less extensive than with 7-day administration of SC azacitidine [11], but extended CC-486 dosing schedules—taken for 14 or 21 days per cycle—were associated with sustained hypomethylation through the end of the treatment cycle [12, 13].

CC-486 administered in extended dosing schedules is currently under investigation in ongoing clinical studies, including two large, randomized phase III trials, as maintenance therapy for patients with AML in first remission after induction chemotherapy (NCT01757535), and as front-line treatment for patients with lower-risk MDS with concurrent thrombocytopenia and RBC transfusion dependence (NCT01566695). In the AML and MDS phase III trials, CC-486 300 mg is administered once-daily (as two 150-mg immediate-release tablets) for 14 or 21 days, respectively, of 28-day cycles.

Studies have shown higher adherence and decreased healthcare resource utilization with single-tablet medication regimens compared with regimens that require multiple pills [15, 16]. Thus, a new formulation of CC-486 was developed that allows for CC-486 to be administered as one 300-mg tablet. This formulation, as a single tablet, would be preferable for use by patients.

We conducted a two-stage, phase I study in adult patients with cancer to evaluate the bioequivalence of the new single 300-mg tablet with that of the two 150-mg tablets used in the two phase III trials. This tablet has different excipients and a different ratio of active pharmaceutical ingredient (API) to excipients compared with the 150-mg tablet. Additionally, because food restrictions can alter PK characteristics, we determined the food effect on the bioavailability of the 300-mg tablet.

## Materials and methods

This phase I, open-label, multicenter, randomized, crossover study was conducted in accordance with Good Clinical Practice, and adhered to the International Conference on Harmonization Guideline E6 and ethical principles outlined in the Declaration of Helsinki. The study received approval from relevant independent review boards or ethics committees (listed in the Supplementary Appendix) before commencement. All patients provided written informed consent. This study is registered at ClinicalTrials.gov (NCT01519011).

### Patients

Eligible patients were age ≥ 18 years with hematologic malignancies or solid tumors and Eastern Cooperative Oncology Group (ECOG) performance status scores ≤ 2, who were relapsed or proved refractory to prior therapy, or for whom no standard treatments were available. Patients with gastrointestinal tumors or tumors that originated or metastasized to the liver were excluded.

### Study design

The trial comprised two stages:

Stage 1 evaluated the relative bioequivalence of the pharmacokinetic (PK) parameters two formulations of CC-486. Patients were randomized 1:1 to receive one dose of CC-486 either as two 150-mg tablets (Formulation A) or a single 300-mg tablet (Formulation B) in a fasted state on day 1, and then crossed over to receive one dose of the alternate CC-486 dosing regimen (also in a fasted state) after an interval of ≥ 48 h following the initial dose.

Stage 2 assessed the food effect on the bioavailability of CC-486 Formulation B. Patients were randomized 1:1 to receive the single CC-486 300-mg tablet on each of two PK study days under fed or fasted conditions, and then crossed over to receive the same 300-mg tablet formulation under the opposite (fasted or fed) condition after an interval of ≥ 48 h.

Fasted state required an overnight fast of at least 8 h, and no food was allowed for at least 2 h post-dose. Water could be taken as desired, except for 1 h before or after CC-486 administration. In the fed state, following an overnight fast, patients were to eat breakfast 30 (± 5) min before planned administration of CC-486. A high-fat (~ 50% of total caloric content) and high-calorie (~ 800–1000 cal) breakfast was consumed, comprising approximately 150, 250, and 500–600 cal from protein, carbohydrate, and fat, respectively.

Blood samples for PK assessments were collected before administration of CC-486, and at 0.25, 0.5, 1, 1.5, 2, 2.5, 3, 3.5, 4, 6, and 8 h post-dose. All plasma PK samples were analyzed centrally using a validated proprietary high-performance liquid chromatography/tandem mass spectrometric method. The lower limit of quantitation for azacitidine in human plasma was 1.00 ng/mL, with linearity demonstrable to 1000 ng/mL (upper limit of quantitation). PK parameters evaluated were area under the plasma concentration–time curve from time 0 to time *t* (time to last measurable concentration [AUC_*t*_]) and from time 0 extrapolated to infinity (AUC_*∞*_), maximum plasma concentration (*C*_max_), time to *C*_max_ (*T*_max_), terminal half-life (*t*_1/2_), apparent total clearance (CL/*F*) and volume of distribution (Vz/*F*).

After completing Stage 1 or Stage 2, patients could enter an extension phase of the study and receive azacitidine 75 mg/m^2^/day IV or SC for 7 consecutive days of repeated 28-day cycles for up to six cycles.

### Statistical analyses

We chose a sample size of 54 patients in each stage (approximately 60 patients enrolled to account for ~ 10% dropout rate) to provide 90% power to conclude bioequivalence and food effect, respectively, assuming the within-patient coefficient of variation as approximately 30%. Power calculations are based on a two one-sided test procedure at the 5% significance level for bioequivalence acceptance limits (80, 125) and assumes a true mean ratio of the log-transformed PK parameters AUC_*∞*_ and *C*_max_ between 0.95 and 1.05 when comparing the test (1 × 300 mg tablet) and reference (2 × 150 mg tablet) in the bioequivalence stage; and the test (1 × 300 mg tablet under fasted condition) and reference (1 × 300 mg tablet under fed condition) in the food effect stage. For *T*_max_, a non-parametric statistical method was used to compare the median *T*_max_ between formulations A and B, and between fed vs fasted states.

## Results

### Bioequivalence (Stage 1)

Although planned to enroll 54 patients, preliminary analysis of data from the first 30 patients enrolled in Stage 1 demonstrated bioequivalence between the two formulations. Among these patients, median age was 68.5 years (range 46–86). Three patients (10%) had hematologic malignancies and 27 (90%) had solid tumors (Supplementary Table 1). Most patients were white (90%), male (67%), and had previously received anticancer or immunomodulatory agents (90%).

**Fig. 1:**
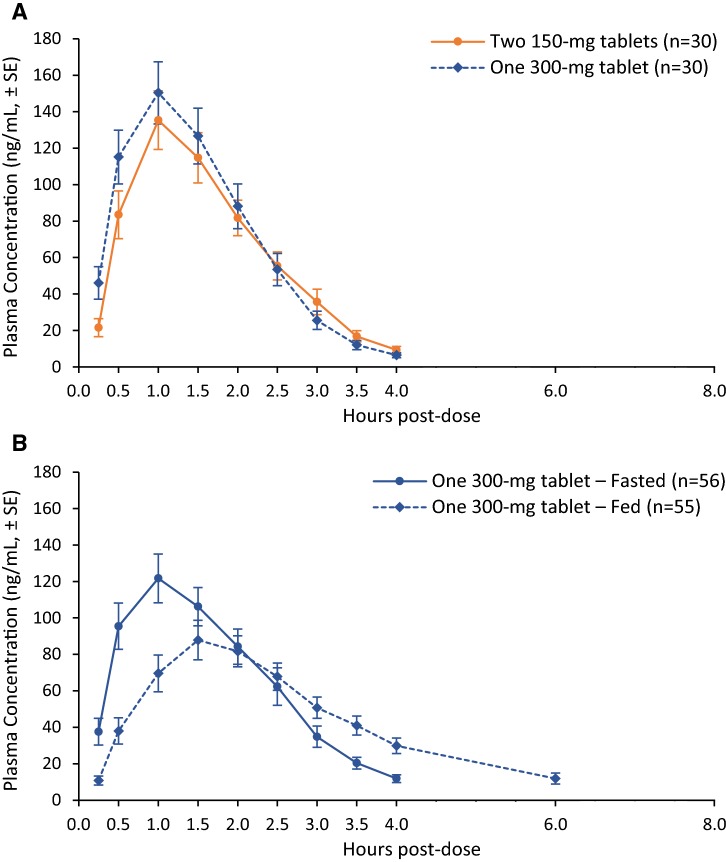
**a** Bioequivalence: arithmetic mean (± SE) CC-486 plasma concentration–time profiles for two CC-486 formulations (Formulation A, 2 × 150 mg; Formulation B, 1 × 300 mg). **b **Food effect: arithmetic mean (± SE) CC-486 plasma concentrations with one 300-mg tablet (Formulation B) in fasted and fed states

CC-486 was rapidly absorbed following administration of a single 300-mg oral dose. Mean plasma concentration–time profiles were well characterized over the sampling interval and were below the level of detection from the 6 h post-dose timepoint (Fig. 1a). For Formulation A and Formulation B, mean *C*_max_ values were 143.0 and 145.1 ng/mL, respectively, and mean AUC_*∞*_ values were 228.5 and 241.6 ng*h/mL. The mean *t*_½_ was 0.544 and 0.492 h, mean CL/*F* was 1313 and 1242 L/h, and mean Vz/*F* was 1031 and 881.1 L for Formulation A and Formulation B, respectively. For both formulations, a median *T*_max_ of 1.0 h was observed with no statistically significant difference (*P* < 0.710) (Supplementary Table 2). The ratio of the geometric means of *C*_max_ was 101.5% (90% CI 89.9, 114.7) and of total drug exposure (AUC_*∞*_) was 105.7% (95.0, 117.6), indicating Formulation B is bioequivalent to Formulation A (Supplementary Table 2).

### Food effect (Stage 2)

A total of 59 patients were enrolled in the food effect portion of this study. Two patients discontinued before receiving study drug, so 57 patients were used to determine the effect of food on the bioavailability of CC-486 Formulation B. Median age was 62 years (range 31–88). All 57 patients had solid tumors (Supplementary Table 1). As in Stage 1, most patients were white (93.0%), male (59.6%), and had received prior anticancer or immunomodulatory agents (94.7%).

Following administration of a single oral dose of 300 mg under fasted and fed conditions, CC-486 was rapidly absorbed and mean plasma concentration–time profiles were well characterized over the sampling interval and were below the level of detection between 6 and 8-h post-dose sampling time points (Fig. 1b). Under fasted and fed condition, respectively, mean *C*_max_ was 131.7 and 105.2 ng/mL, mean AUC_*∞*_ was 234.5 and 248.9 ng*h/mL, mean *t*_½_ was 0.58 and 0.78 h, mean CL/*F* was 1280 and 1205 L/h, and mean Vz/*F* was 1074 and 1279 L (Table 1).

**Table 1:** Food effect on the plasma pharmacokinetic parameters of the CC-486 300 mg Formulation B tablet

Condition	Statistic	AUC_*t*_ (ng*h/mL)	AUC_*∞*_ (ng*h/mL)	*C*_max_ (ng/mL)	*T*_max_ (h)	*t*_1/2_ (h)	CL/*F* (L/h)	Vz/*F* (L)
Fasted	*N*	56	54	56	56	54	54	54
	Geometric mean*	224.9	234.5	131.7	NA	0.58	1280	1074
	Geometric %CV	68.4	62.9	70.4	NA	31.6	62.9	76.2
	Median	240	243	141	1.0	0.55	1230	1140
	Min, max	41.5, 1050	65.9, 1050	21.4, 608	0.50, 4.0	0.35, 1.7	286, 4550	228, 6310
Fed	*N*	55	44	55	55	46	44	44
	Geometric mean*	233.7	248.9	105.2	NA	0.78	1205	1279
	Geometric %CV	58.3	52.0	76.3	NA	55.2	52.0	80.5
	Median	250	250	116	2.0	0.68	1200	1160
	Min, max	61.3, 554	78.9, 560	22.7, 337	0.50, 6.1	0.40, 6.2	536, 3800	399, 9350

PK parameter	Condition	*N*	Geometric mean^†^	Ratio (%) of geometric means	90% CI of ratio (%) of geometric means	Intra-patient %CV
AUC_*t*_ (ng*h/mL)	Fasted	56	227.8	101.7	(92.83, 111.5)	29.0
	Fed	55	231.8			
AUC_*∞*_ (ng*h/mL)	Fasted	56	133.0	108.9	(98.48, 120.5)	28.3
	Fed	55	104.9			
*C*_max_ (ng/mL)	Fasted	56	133.0	78.86	(68.58, 90.68)	45.5
	Fed	55	104.9			

PK parameter	Condition	*N*	Median	Median difference	90% CI of median difference (fed − fasted)	*P* value
*T*_max_ (h)^‡^	Fasted	56	1.0	1.0	(0.75, 1.3)	< 0.001
	Fed	55	2.0			

AUC_*∞*_, *t*_1/2_, CL/*F*, and *V*/*F* could not be calculated in some instances, if there were not data from a sufficient number of time points after the occurrence of *C*_max_. Slight differences in geometric means of AUC_*∞*_ and *C*_max_ are due to different methods of calculation

*N patients* the total number of patients for which the PK parameter could be calculated, *AUC*_*t*_ area under the plasma concentration–time curve from time 0 to time *t*, *AUC*_*∞*_ area under the plasma concentration–time curve from time 0 extrapolated to infinity, *C*_*max*_ maximum plasma concentration, *T*_*max*_ time to *C*_max_, *t*_*1/2*_ terminal half-life, *CL/F* apparent total clearance, *Vz/F* volume of distribution, *NA* not applicable

*Calculated using summary statistics

†Calculated using an analysis of variance (ANOVA) model

^**‡**^Median and median difference (test vs. reference), and 90% CI of the median difference, are from Hodges–Lehmann estimate. The *P* value is from Wilcoxon signed-rank test.

The 90% CI of the ratio of the geometric means for *C*_max_ (68.6–90.7%) did not include 100%, indicating that there was a statistically significant difference for *C*_max_ (Table 1), with a fed/fasted ratio of the geometric means of approximately 79%. Moderate intra-patient variability was noted for *C*_max_ (CV% = 45.5%). Consistent with an effect of food on gastric emptying, median *T*_max_ was reached at 2 h and 1 h postdose in fed and fasted states, respectively (*P* < 0.001) (Table 1).

Despite differences in *C*_max_ and *T*_max_, overall drug exposure (AUC_*∞*_) in fasted and fed states was similar: the fed/fasted ratio of the geometric means was 108.9% and the 90% CI of the ratios of the geometric means for AUC_*∞*_ (90% CI 98.5, 120.5) were within the 80–125% bioequivalence limits (Table 1). CL/*F* and Vz/*F* were also comparable in fed and fasted states. Again, moderate to high interpatient variability was observed for all CC-486 PK parameters.

## Discussion

Oral antineoplastic agents are a primary form of treatment in many malignancies. When efficacy and safety are not compromised, oral agents are generally preferred to parenteral treatments [17]. Compared with parenteral therapy, oral anticancer agents allow convenient dosing outside of the clinic [17]. However, adherence is required to ensure therapeutic efficacy and to avoid compromising treatment outcomes, especially in cases of symptomatic or rapidly progressing disease, where dose-intensity is important [18]. Indeed, suboptimal adherence may be the greatest barrier to effective use of oral anticancer agents [19]. Studies show statistically significant correlations between medication nonadherence and clinical and resource utilization outcomes, including cancer progression, prolonged inpatient durations, higher total healthcare spending, and poorer survival [20].

The simplicity of the anticancer regimen may influence adherence to treatment. Many patients with cancer are older and may have multiple comorbidities for which they take multiple medications. The number of anticancer pills and frequency of administration can add to the medication burden that patients experience [21] and adherence rates tend to be higher when simpler, once-daily regimens are combined with lower pill burden [16]. This study showed that a single oral CC-486 Formulation B 300-mg tablet was bioequivalent to two Formulation A 150-mg tablets. CC-486 is intended for once-daily dosing. Theoretically, a single tablet prohibits dose splitting, increasing the likelihood that patients receive the correct dose of the prescribed medication.

A lack of food restrictions may also enhance adherence to drug therapy. In this study, food was shown to have no clinically relevant effect on PK parameters of the 300-mg tablet compared with a fasted condition. Although *C*_max_ occurred later and was decreased by ~ 21% after a high-fat meal compared with the fasted state, systemic drug exposure (AUC) in fed and fasted states was within the bounds of bioequivalence (80–125%). Thus, observed differences in *C*_max_ and *T*_max_ between fed and fasted states would not be expected to have a clinical impact. These results are generally consistent with outcomes of an early PK study of Formulation A (two 150-mg tablets) that showed no effect of food on PK parameters (*C*_max_ and AUC_*∞*_) compared with the fasted state [22]. Unlike the earlier study, however, the current study evaluated food effect on a single 300-mg tablet after a high-fat meal (per FDA guidance), and was statistically powered to make more definitive PK comparisons with the fasted state.

The single 300-mg CC-486 Formulation B tablet was found to be bioequivalent to two 150-mg Formulation A tablets, that have been shown efficacious and generally well-tolerated in clinical trials [14], and the 300-mg CC-486 tablet can be taken with or without food. The single-tablet formulation will be more convenient for patients and will be used for registration purposes to support further development of CC-486 for use in various malignancies.

## Electronic supplementary material

Below is the link to the electronic supplementary material.

Supplementary file1 (PDF 183 kb)
