# Supplementary material for: Evaluation of the bioequivalence and food effect on the bioavailability of CC-486 (oral azacitidine) tablets in adult patients with cancer
Source: Cancer Chemother Pharmacol. 2020 Feb 8;85(3):621–6. doi: 10.1007/s00280-020-04037-9 (PMC7036073; doi:10.1007/s00280-020-04037-9)
Supplement: Supplementary file 1 — Supplementary file1 (PDF 183 kb) [file 280_2020_4037_MOESM1_ESM.pdf]

SUPPLEMENTAL FILE

Evaluation of the Bioequivalence and Food Effect on the Bioavailability of CC-486 (Oral Azacitidine) Tablets in Adult Patients with Cancer

Hani M. Babiker, MD; Mohammed Milhem, MBBS; Joseph Aisner, MD; William Edenfield, MD; Dale Shepard, MD, PhD; Michael Savona, MD; Swaminathan Iyer, MD, PhD; Maen Abdelrahim, MD, PhD; CL Beach, PharmD; Barry Skikne, MD; Eric Laille, MS; Kao-Tai Tsai, PhD; Thai Ho, MD, PhD

Journal: *Cancer Chemotherapy and Pharmacology*

Corresponding author: Hani M. Babiker, M.D.
University of Arizona Comprehensive Cancer Center
hanibabiker@email.arizona.edu

Ethics Committees and Institutional Review Boards for the CC-486-CAGEN-001 Study

Site No.	Investigator Name	Name/Address of IEC/IRB	Name of Chairperson(s)
001	William Edenfield	Greenville Health System (GHS)<br>Institutional Review Board, Committee C<br>701 Grove Road<br>Greenville, SC 29605	James Hayes, MD
002	Vivek Khemka	Western Institutional Review Board – WIRB<br>1019 39th Avenue SE, Suite 120<br>Puyallup, WA 98374	Erin Brower, MS, CIP
005	Thai Ho	Mayo Clinic Institutional Review Board<br>200 First Street SW<br>Rochester, MN 55905	Ouellette Yves, MD
007	Michael Savona	Vanderbilt University Institutional<br>Review Board<br>1313 21st Avenue South<br>504 Oxford House<br>Nashville, TN 37232-4315	James A.S. Muldowney, MD
009	Maen Abdelrahim	The Methodist Hospital Research<br>Institutional Review Board<br>1130 John Freeman<br>MS: MJG6-014<br>Houston, TX 77030<br>Former Address:<br>The Methodist Hospital Research<br>Institutional Review Board<br>6565 Fannin Street<br>Houston, TX 77030	Susan Miller, MD, MPH

Supplementary Table 1. Baseline demographic and disease characteristics

	Bioequivalence Cohort (Stage 1) (N = 30)	Food Effect Cohort (Stage 2) (N = 57)
**Age**, median (range)	68.5 (46, 86)	62.0 (31, 88)
**Race**, n (%)		
White	27 (90)	53 (93)
Other[a]	2 (6)	2 (4)
Not Collected or Reported	1 (3)	2 (4)
**Sex**, n (%)		
Male	20 (67)	34 (60)
Female	10 (33)	23 (40)
**BMI**, median (range)	27.8 (16, 44)	26.6 (19, 47)
**Malignancy type**, n (%)		
Metastatic or inoperable solid tumors	27 (90)	57 (100)
Myelodysplastic syndromes	1 (3)	0
Acute myeloid leukemia	1 (3)	0
Multiple myeloma	1 (3)	0
**Time since diagnosis (years)**, median (range)	3.5 (0.1, 19.2)	4.8 (0.2, 27.1)
**ECOG performance status score**, n (%)		
0	7 (23)	20 (35)
1	23 (77)	34 (60)
2	0	3 (5)

[a]Black or African-American, Asian, American Indian or Alaskan Native

BMI, body mass index; ECOG, Eastern Cooperative Oncology Group.

Supplementary Table 2. Plasma pharmacokinetic parameters for two formulations of CC-486 (2 x 150-mg tablets [Formulation A] and 1 x 300-mg tablet [Formulation B])

Treatment	Statistics	AUCt (ng*h/mL)	AUC$_{\infty}$ (ng*h/mL)	C_{max} (ng/mL)	T_{max} (h)	$t_{1/2}$ (h)	CL/F (L/h)	Vz/F (L)
**2 x 150 mg tablets**	N	30	30	30	30	30	30	30
	Geometric Mean	225.0	228.5	143.0	NA	0.544	1313	1031
	Geometric %CV	63.7	62.6	53.1	NA	32.0	62.6	67.4
	Median	226	232	149	1.0	0.52	1300	922
	Min, Max	71.8, 555	76.4, 557	34.5, 362	0.48, 3.0	0.34, 1.1	538, 3930	389, 3860
**1 x 300 mg tablet**	N	30	30	30	30	30	30	30
	Geometric Mean	239.1	241.6	145.1	NA	0.492	1242	881.1
	Geometric %CV	65.2	64.5	63.7	NA	26.9	64.5	67.4
	Median	236	237	148	1.0	0.46	1270	900
	Min, Max	93.0, 640	94.5, 642	46.7, 362	0.50, 2.5	0.33, 0.87	468, 3170	317, 2790

PK Parameter	Treatment	N	Geometric Mean	Ratio (%) of Geometric Means	90% CI of Ratio (%) of Geometric Means	Intra-patient %CV
**AUCt (ng*h/mL)**	2 x 150 mg<br>1 x 300 mg	30<br>30	225.0<br>239.1	106.27	(95.21, 118.62)	25.4
**AUC$_{\infty}$ (ng*h/mL)**	2 x150 mg<br>1 x 300 mg	30<br>30	228.5<br>241.6	105.71	(95.01, 117.62)	24.7
**C_{max} (ng/mL)**	2 x 150 mg<br>1 x 300 mg	30<br>30	143.0<br>145.1	101.52	(89.87, 114.68)	28.3

PK Parameter	Condition	N	Median	Median Difference	90% CI of Median Difference (Fed – Fasted)	*P*-value
**T_{max} (h)***	Fasted<br>Fed	30<br>30	1.0<br>1.0	-0.017	(-0.25, 0.03)	<0.710

N patients = the total number of patients for which the PK parameter could be calculated

*Median and median difference (Test vs. Reference), and 90% CI of the median difference, are from Hodges-Lehmann estimate. The P value is from Wilcoxon signed-rank test.

AUC$_{\infty}$, area under the plasma concentration-time curve from time 0 extrapolated to infinity; C_{max}, maximum plasma concentration; T_{max}, time to C_{max}; $t_{1/2}$, terminal half-life; CL/F, apparent total clearance; Vz/F, volume of distribution; NA, not applicable.
